# Host Cell Copper Transporters CTR1 and ATP7A are important for Influenza A virus replication

**DOI:** 10.1186/s12985-016-0671-7

**Published:** 2017-01-23

**Authors:** Jonathan C. Rupp, Manon Locatelli, Alexis Grieser, Andrea Ramos, Patricia J. Campbell, Hong Yi, John Steel, Jason L. Burkhead, Eric Bortz

**Affiliations:** 1 Department of Biological Sciences, University of Alaska Anchorage, Anchorage, AK USA; 2 Department of Microbiology and Immunology, Emory University School of Medicine, Atlanta, Georgia USA; 3 Present address: Institute Albert Bonniot – INSERM U1209, Université Grenoble Alpes, Grenoble, France

**Keywords:** Copper, Copper transport, ATP7A, CTR1, Influenza virus, Cell metabolism

## Abstract

**Background:**

The essential role of copper in eukaryotic cellular physiology is known, but has not been recognized as important in the context of influenza A virus infection. In this study, we investigated the effect of cellular copper on influenza A virus replication.

**Methods:**

Influenza A/WSN/33 (H1N1) virus growth and macromolecule syntheses were assessed in cultured human lung cells (A549) where the copper concentration of the growth medium was modified, or expression of host genes involved in copper homeostasis was targeted by RNA interference.

**Results:**

Exogenously increasing copper concentration, or chelating copper, resulted in moderate defects in viral growth. Nucleoprotein (NP) localization, neuraminidase activity assays and transmission electron microscopy did not reveal significant defects in virion assembly, morphology or release under these conditions. However, RNAi knockdown of the high-affinity copper importer CTR1 resulted in significant viral growth defects (7.3-fold reduced titer at 24 hours post-infection, *p* = 0.04). Knockdown of CTR1 or the *trans*-Golgi copper transporter ATP7A significantly reduced polymerase activity in a minigenome assay. Both copper transporters were required for authentic viral RNA synthesis and NP and matrix (M1) protein accumulation in the infected cell.

**Conclusions:**

These results demonstrate that intracellular copper regulates the influenza virus life cycle, with potentially distinct mechanisms in specific cellular compartments. These observations provide a new avenue for drug development and studies of influenza virus pathogenesis.

## Background

Influenza A remains a critical concern not only for human health but also for wildlife health and the livestock industry. Seasonal human strains cause significant mortality [1], and highly pathogenic avian viruses result in flock loss as well as continuing to threaten new human pandemics [2, 3]. While vaccination of human populations is one available intervention, it is not completely effective and may not prevent the emergence and spread of novel viruses. Application of antiviral therapies is another approach to increase our readiness for pandemic outbreaks. Antivirals such as oseltamivir, which target viral processes, have shown utility, but drug resistant viruses can emerge [4]. Antivirals that target host processes important for the virus have potential to circumvent the development of resistance, and targeting of host processes has shown therapeutic promise for other viruses [5]. Several specific host factors important for influenza replication have been identified, for example; the endosomal coat protein complex (COP-I) and vacuolar ATPase regulate virion entry and uncoating [6], RNA binding proteins are essential for viral RNA synthesis [7], and karyopherins are involved in nucleocytoplasmic transport of viral ribonucleoprotein (vRNP) [8]. Likewise, some host processes that regulate influenza A virus budding and release have been identified. The F1Fo-ATPase was found to be important for release of infectious virus particles [9], while in contrast, virion release for some influenza A virus strains is antagonized by histone deacetylase 6 [10] and the antiviral protein tetherin [11]. Discovery of additional host processes involved in virion production will provide additional options for therapy development.

Copper handling machinery is evolutionarily conserved throughout eukaryotes, and copper homeostasis is recognized as important in human health. Copper transport machinery has been identified as important in macrophage antimicrobial responses [12]; however, little is known about the role of copper related processes in influenza infection. Copper is an important cofactor for a number of critical cellular processes, including cellular respiration and mitigation of oxidative stress. Many additional copper-dependent processes are observed in specific tissues, such as modulation of lipid metabolism in the liver [13, 14], and neurotransmitter processing in the brain [15, 16]. In the lung and respiratory tract, extracellular matrix synthesis requires the copper-containing enzyme lysyl oxidase [17]. Recent work has further revealed altered expression of copper transporters in response to pulmonary hypertension [18]. As copper ions exist in two oxidative states, reduced Cu^1+^ (I) and oxidized Cu^2+^ (II), with different biological activities, the cell maintains tight control of copper oxidation and subcellular distribution through regulation of highly conserved copper binding proteins and transport machinery (for review, see [19]). This control is mediated by a system of copper transporters and chaperones that direct copper ions, generally monovalent Cu (I), to cellular compartments for physiological functions. The copper transporter CTR1 (SLC31A1) is important for uptake of copper ions, imported as Cu (I), from the extracellular space [20]. CTR1 is expressed in most tissues, and defects may be related to disease conditions including Alzheimer’s disease [21]. The copper transporter ATP7A is also expressed in most cells, and transports Cu (I) ions from the cytosolic compartment into the *trans*-Golgi network, vesicles, and eventually exports excess copper into the extracellular space; defects in this gene are the cause of Menkes disease [22]. ATP7A’s subcellular location is modified in response to different conditions, which is one mechanism in the maintenance of copper homeostasis [23].

Little is known about the role of copper and copper dependent processes in influenza-infected cells. In an RNAi based screen for host factors, knockdown of several genes that regulate copper homeostasis including CTR1 and ATP7A was reported to affect influenza virus replication in human lung A549 cells [6], suggesting a potential role for copper in viral replication. Additionally, studies have found that copper can inactivate avian influenza particles on surfaces and clothing [24, 25], and the copper dependent enzyme SOD1 is important for influenza oxidative stress regulation [26]. Further, the viral ion channel M2 was found to be inhibited by copper ions in an oocyte based experimental system [27]. There are also some data that indicate an effect of dietary copper on immune response to influenza [28], which supports further investigation into the role of copper in influenza infection. Perhaps the most telling research on the effect of intracellular copper on the virus showed that thujaplicin-copper chelates inhibited influenza induced apoptosis and viral particle production in a tissue culture (MDCK cells) model of infection [29].

To further investigate the influence of cellular copper on influenza A virus replication, we tested the effect of altered copper environments on the viral life cycle in a tissue culture model of lung cell infection. We assessed the effects on the virus that result from altering the amount of copper available to the cells, as well as the effects from knocking down host genes related to copper homeostasis. We evaluated the impact these treatments had on virus replication generally, as well as on specific aspects of viral function. We observed that altered copper concentration in the growth medium and knockdown of host gene expression resulted in distinct viral replication defects. These results begin to define the importance of cellular copper metabolism in influenza processes, and indicate the copper related pathways that show promise for further investigation.

## Methods

### Cultured cells and virus

A549 lung adenocarcinoma cells and Madin-Darby Canine Kidney (MDCK) cells were cultured in DMEM (Corning Inc., Manassas, VA) supplemented with 10% FBS (Atlas Biologicals, Fort Collins, CO).

Influenza A/WSN/33 (H1N1) virus stocks were grown in MDCK cells, and titered by plaque assay on MDCK cells [7]. The genome of our stock of WSN was sequenced and compared to those recorded in the Influenza Research Database. Two point mutations were identified in our stock, which are not present in the deposited sequences. In segment 6 (NA), a G to U change at position 158 of the ORF results in a Ser to Ile change at residue 53. In segment 7 (M), an A to G change at position 502 of the ORF results in a Thr to Ala change at residue 168. This latter change is known [30] and does not alter virus growth characteristics in cultured MDCK cells.

### Copper and chelator treatments

Cells were treated with 50 μM CuCl_2_ (Acros Organics, Morris Plains, NJ) or 10 μM ammonium tetrathiomolybdate (TTM; Sigma-Aldrich, St. Louis, MO) by supplementing normal growth medium and inoculums, beginning at 24 hours prior to subsequent treatments, *i.e.* infection. TTM is an efficient intracellular copper chelator [31, 32]. Intracellular copper concentrations in complete lysates of untreated, 10 μM TTM, and 50 μM CuCl_2_ treatment of A549 cells were assessed by inductively coupled plasma mass spectrometry (ICP-MS) elemental analysis (courtesy of M. Ralle, Oregon Health & Science University). Cytotoxicity of CuCl_2_ and TTM on cell viability was assayed by chemiluminescent ATP quantitation; CellTiter-Glo (Promega, Madison, WI). No decrease in luminescence was observed below concentrations of CuCl_2_ or TTM at least 5 fold higher than used for this study. Additionally, the possible effect of these treatments on virion viability was assayed. Copper ions have previously been seen to inactivate H9N2 virions [24]. To determine if such inactivation was occurring in our conditions, inoculums were prepared as for infections and incubated in the presence of CuCl_2_ or TTM but without cells. No effect on titer was observed at the concentrations used for this study.

### RNAi knockdowns

Expression of cellular copper transport genes in A549 cells was reduced by transfection with endoribonuclease-prepared siRNAs (esiRNAs). Transfection mixes were prepared with Lipofectamine RNAiMax (Life Technologies, Carlsbad, CA) and 5 to 20 nM of siRNA Universal Negative Control, MISSION esiRNA human CTR1 (SLC31A1), or MISSION esiRNA human ATP7A (Sigma-Aldrich, St. Louis, MO). Cells were seeded onto mixes 36 hours prior to infection.

MISSION esiRNAs (Sigma-Aldrich) comprise a multiplex pool of siRNA that target a specific mRNA sequence, leading to highly specific gene silencing [33]. The effect of knockdowns on cell viability was assessed, as for CuCl_2_ or TTM treatments above, by CellTiter-Glo. Experimental esiRNA concentrations were chosen such that cell viability, as determined by this assay, was equivalent to the negative control siRNA knockdown. Knockdown efficiencies were validated by quantitative reverse-transcriptase–PCR (qRT-PCR) with primers specific to the target gene. For both esiRNAs, the target transcript levels were reduced by around 90% relative to the negative control siRNA knockdown (data not shown).

### Viral RNA quantification

Control A549 cells and those treated with either Cu, TTM or esiRNA were infected at multiplicity of infection (MOI) = 1, and at the indicated times were washed with phosphate buffered saline (PBS). Lysates were harvested in buffer RLT and RNAs extracted by RNeasy kit (Qiagen, Valencia, CA). Viral RNA was quantified by qRT-PCR, using SYBR green based detection. Reverse-transcription and PCR reactions were performed in one tube with the iTaq kit (BioRad, Hercules, CA), in a BioRad CFX96 thermocycler. Primers for the viral RNA were specific to the nucleoprotein (NP) gene (segment 5). Similar results were obtained with primers specific to the M gene (segments 7), thus we present the representative NP data. Primers specific to 18S rRNA were used as the reference, and relative expression was calculated using the 2^(−Delta Delta C(T)) method [34]. Statistical significance was assessed by paired two-tailed *t*-test, *p* < 0.05.

### Viral minigenome assay

Viral polymerase activity was assessed using an experimentally optimized minigenome assay with viral polymerase expression vectors (VPOL: pCAGGS-NP and pCAGGS-PB1, −PB2, and -PA, in a 5:2:1:2 ratio), a vRNA firefly luciferase reporter construct (minigenome), and *Renilla* luciferase expression plasmid as an internal transfection control, as we described previously [7]. A549 cells were transfected with esiRNA and incubated for 36 hours. Cells were then transfected with VPOL, minigenome, and *Renilla* plasmids, using the FuGENE HD transfection reagent (Promega), following the manufacturer’s recommendations. 24 hours after the second transfection, cells were harvested and assayed using the Dual Luciferase Reporter Assay (Promega) on a BioTek Synergy HT reader.

### Viral protein quantification

Proteins were extracted from the same samples harvested for viral RNA quantification, above. Extractions from buffer RLT were performed using the iced acetone method described by the manufacturer (Qiagen). Proteins were separated by denaturing SDS polyacrylamide gel electrophoresis, and transferred to PVDF (Pall Corp., Pensacola, FL). Immunoblotting was performed with monoclonal antbody to influenza NP (AA5H; AbCam, Cambridge, MA) or anti-M1 polyclonal (a kind gift of Dr. Adolfo García-Sastre, Icahn School of Medicine at Mount Sinai), and peroxidase conjugated secondary antiserum. Blots were imaged with Supersignal substrate (ThermoFisher Scientific, Carlsbad, CA), on a Cell Biosciences FluorChem HD2. Consistent loading was monitored by Coomassie Brilliant Blue R-250 (Amresco, Solon, OH) staining of the post-transfer gel.

### Viral growth kinetics and neuraminidase activity

Control A549 cells and those treated with either Cu, TTM or esiRNA were infected at MOI = 1. Culture medium was sampled and replaced at 12-hour intervals. The titer of infectious particles was quantified by immunostaining in MDCK cells as follows: inocula were prepared by tenfold serial dilution of the samples, and subconfluent MDCK monolayers in 96 well plates were infected. After 8 hours, cells were fixed in 4% paraformaldehyde in PBS, and permeabilized with 0.1% NP-40 in PBS. Membranes were blocked with 1% non-fat dry milk, then probed with antiserum to the NP protein and a fluorescently tagged secondary antiserum, and fluorescent foci counted. Total counts of each well were taken, for at least two dilutions per sample. Dilutions showing between 5 and 500 fluorescent foci were chosen, and the fluorescent forming units (FFU) per mL calculated as an average from these multiple counts.

Neuraminidase (NA) activity in the harvested medium was quantified by NA-Fluor kit (Applied Biosystems, Foster City, CA). Serial dilutions of samples were combined with an equal volume of substrate working solution, and incubated 60 minutes. The stop solution was added and fluorescence determined in a BioTek Synergy HT reader. Fluorescence values were normalized to the titer of each sample.

### Immunofluorescence microscopy

CuCl_2_ concentrations were 10 μM for this experiment. Treated A549 cells were infected at MOI = 1. At 12 hours post infection (h.p.i.), cells were washed with PBS, fixed in 4% paraformaldehyde, and permeabilized with 0.1% saponin. Samples were probed with primary antisera using sheep anti-TGN46 (Serotec), rabbit anti-ATP7A (a gift from S. Lutsenko), or anti-NP monoclonal AA5H, in PBS with 0.05% Tween 20 and 3% bovine serum albumin. Secondary antisera conjugated to Alexa Fluor 488, 532, or 647 were used for visualization, and mounted in VectaShield with DAPI (Vector Laboratories, Burlingame, CA). Images were captured at room temperature with a Leica DM6000 B microscope with a 63x oil immersion objective, numerical aperture = 1.4, and a Photometrics (Tucson, AZ) CoolSNAP MYO camera. Software for capture and deconvolution was Leica Application Suite X (LAS X) and image placement Adobe Illustrator.

### Transmission electron microscopy

Treated A549 cells were infected at MOI = 5. At 16 h.p.i., cells were washed with PBS and fixed in 2.5% glutaraldehyde (Electron Microscopy Sciences, Fort Washington, PA) and 0.1 M cacodylate, pH 7.4. Cells were then embedded in Eponate 12 resin, cut into 80-nm sections, and stained with 5% uranyl acetate and 2% lead citrate at the Emory Robert P. Apkarian Integrated Electron Microscopy Core. After sample preparation, grids were imaged at 75 kV using a Hitachi H-7500 transmission electron microscope.

## Results and discussion

Processes in cellular copper metabolism overlap with the influenza virus lifecycle. To study their relationship, if any, we examined the effect of the intracellular copper concentration on influenza A replication. Using the human lung epithelial adenocarcinoma cell line A549; intracellular copper (I) concentration was raised by supplementing the growth medium with 50 μM CuCl_2_, or lowered by supplementing with 10 μM of copper chelator TTM. TTM decreases the bioavailable copper [35], which promotes *trans*-Golgi localization of the copper exporter ATP7A [36]. As expected, intracellular copper concentration relative to protein content in A549 cell extracts was approximately 15-fold higher for 50 μM CuCl_2_ treatment, and 3-fold lower for 10 μM TTM treatment, in comparison to untreated A549 cells, as measured by ICP-MS elemental analysis (Table 1).

**Table 1:** Elemental analysis of total intracellular copper in A549 cells by ICP-MS

A549 Treatment^a^	Protein BCA^b^ ug/ul	Ionized Cu^2+^ ICP-MS^c^ ug/g	Intracellular Cu^2+^ Ratio to Control^d^
Media	0.663	4.0	1.0
10 μM TTM	0.686	1.2	0.3
50 μM CuCl2	0.144	12.9	14.8
Negative Control siRNA	0.680	2.7	1.0
CTR1 esiRNA	0.472	1.6	0.9
ATP7A esiRNA	0.502	18.2	9.1

^a^A549 cells were untreated (Media), treated with 50 μM CuCl_2_ or 10 μM ammonium tetrathiomolybdate (TTM), or transfected with indicated siRNA for 24 hours, washed in PBS and lysed in RIPA buffer

^b^Protein measured by BCA assay

^c^Elemental analysis of total intracellular copper (ionized to Cu^2+^) by inductively coupled plasma mass spectrometry (ICP-MS)

^d^Intracellular Cu was normalized to protein content for control conditions of Media alone (χ^2^ < 0.001), or Negative Control siRNA (χ^2^ < 0.001), expressed as a ratio to each control condition, respectively; χ^2^, chi-squared test against null model

**Fig. 1:**
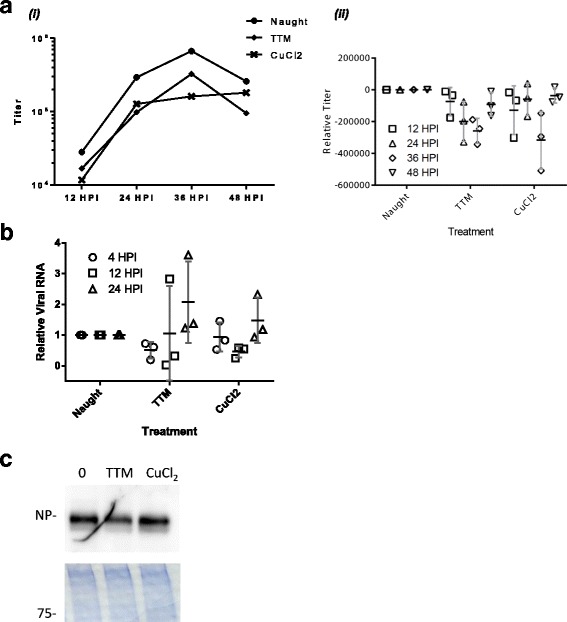
Addition of copper or chelator inhibits viral growth but not macromolecular accumulation. A549 cells’ growth medium was supplemented with 50 μM CuCl_2_ or 10 μM TTM, to alter intracellular copper concentration, 24 hours before infection with influenza A/WSN/33 (H1N1). (**a**) At the indicated times post infection, infectious particles released into the medium were determined by immunostain assay. A representative data set is shown *(i)*, with mean and standard deviation from the control condition for 3 independent experiments shown in accompanying panel *(ii)*. Titer = fluorescent center forming units per mL. (**b**) At the indicated times cells were lysed and RNA extracted. Relative viral RNA amounts were determined by qRT-PCR with primers for the NP (segment 5) RNA and the host 18S rRNA. The means of two technical replicates are displayed as points, with the mean and standard deviation of three biological replicates indicated with whiskers. (**c**) At 12 h.p.i. cells were lysed and proteins extracted. Proteins were subjected to western blotting using antisera to the viral NP protein. A representative blot of at least 3 independent experiments is shown. After transfer the gels were stained with coomassie blue; a section is shown as loading control

Treated cells were then infected with influenza A/WSN/33 (H1N1). Viral growth in cells with altered copper levels was assessed by measuring infectious particles released at 12 hour intervals (Fig. 1a). Alteration of physiological copper concentration in A549 cells resulted in a moderate reduction in the titer of virus produced late in the virus lifecycle, both under 10 μM of chelator TTM at 24 hours post infection (h.p.i.) (*p* = 0.051) and 36 h.p.i. (*p* = 0.005), and 50 μM of exogenous CuCl_2_ at 36 h. p.i. (*p* = 0.038) treatment (Fig. 1a). These data suggest that the homeostatic balance of copper ions in host cells is important in the viral life cycle. To further assess this effect, viral RNA accumulation was assayed in treated cells early and late in infection (Fig. 1b). In this assay, increased copper did not have a significant effect on viral RNA levels. TTM chelator treatment did display a trend of lower RNA levels at 12 h.p.i. (*p* = 0.09), but differences in RNA levels were not significant at later time points or for CuCl_2_ treatment. Thus in both treatments the level of viral RNA in treated cells weakly correlated with the decreased titers observed. To assess effects on viral protein levels, treated cells were harvested at 12 h.p.i. and viral nucleoprotein (NP) levels assessed by western blotting. While less NP protein accumulated under TTM chelator treatment (Fig. 1c), similar to the trend observed with viral RNA synthesis (Fig. 1b), the difference paralleled a significant reduction in infectious titer (Fig. 1a). Viral macromolecular synthesis or accumulation appears less affected than infectious particle production, suggesting that copper-binding host proteins in the cell retain their copper under TTM or CuCl2 treatment, and function relatively normally in the early stages of infection. Thus, the defect caused by altering cellular copper with TTM or CuCl_2_ affects macromolecular synthesis, and in part, efficient assembly and release of new particles. Additionally, fluorescent centers appeared to cluster more in virus preparations from treated cells, an observation that also supports an assembly phenotype (data not shown).

**Fig. 2:**
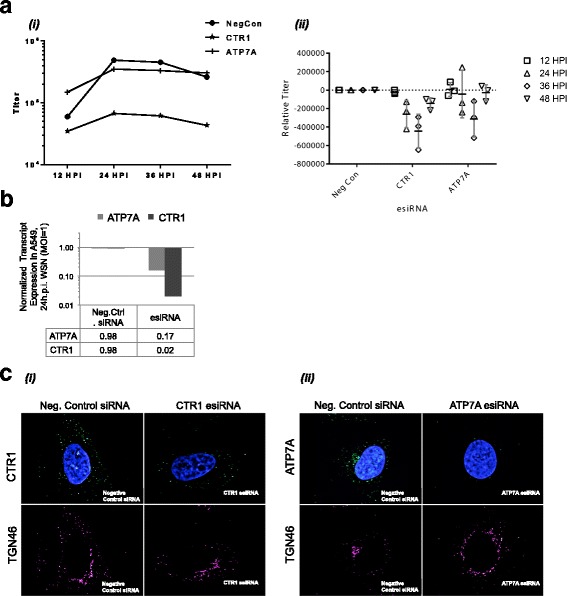
Knockdown of host copper homeostasis genes affects viral growth. A549 cells were transfected with esiRNAs 48 hours before infection with influenza A/WSN/33 (H1N1). (**a**) At the indicated times post infection, infectious particles released into the medium were determined by immunostain assay. A representative data set is shown *(i)*, with mean and standard deviation from the control condition for 3 independent experiments shown in accompanying panel *(ii)*. Titer = fluorescent center forming units per mL. (**b**) Total cellular RNA was harvested, and ATP7A and CTR1 transcripts quantified by qRT-PCR and normalized to 18S rRNA reference by ΔΔCt method. (**c**) ) Immunofluorescence microscopy evaluating knockdown depletion and subcellular localization of ATP7A and CTR1 in uninfected A549 cells; DAPI, blue; TGN46, magenta; CTR1, green *(ii)*; ATP7A, green *(ii)*. Scale bar, 10 μm

To further assess the effect of copper metabolic pathways on influenza infection, we examined the requirements for genes central to copper homeostasis by RNAi knockdown. Viral replication was assayed in cells transfected with endoribonuclease-prepared siRNA (esiRNA) pools [33] to ablate transcripts encoding the copper importer CTR1 or copper transporter ATP7A. In A549 cells targeted by RNAi, intracellular copper was 9-fold higher in ATP7A knockdown (Table 1), consistent with impaired Cu efflux through the secretory pathway; CTR1 knockdown only mildly decreased total intracellular Cu, although intracellular copper distribution could not be assessed. Knockdown of ATP7A resulted in mildly depressed virus production (1.4-fold decrease, *p* = 0.05) only late in infection (36 h.p.i.), However, CTR1 knockdown resulted in marked decrease in infectious particles released by 24 h.p.i. (7.3-fold, *p* = 0.04,) with significant reduction persisting at 36 h.p.i. (*p* = 0.013) (Fig. 2a). CTR1 knockdown exhibited a mild but not significant decrease in titer early in infection (12 h.p.i., *p* = 0.13). Efficiency of knockdown of ATP7A and CTR1 transcripts and protein, in comparison to nontarget siRNA control, were analyzed by quantitative RT-PCR (Fig. 2b) and immunofluorescence assay (Fig. 2c), respectively. These results imply that copper transporter-mediated distribution of intracellular copper is necessary for sustaining efficient viral replication.

**Fig. 3:**
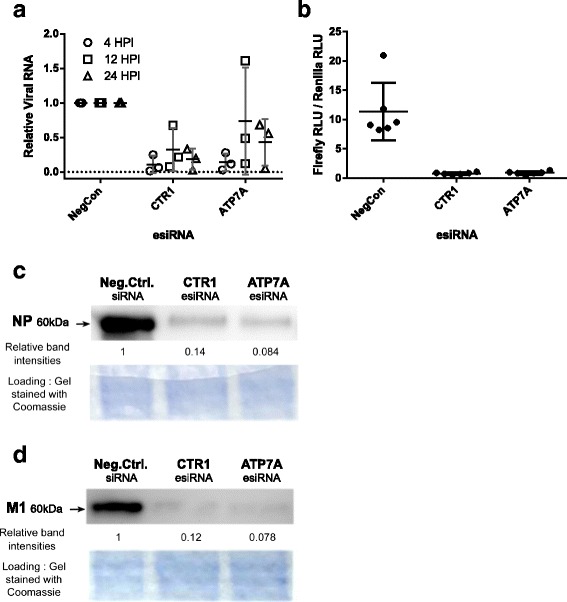
Knockdown of host copper homeostasis genes affects viral macromolecular accumulation. (**a**) At the indicated times cells were lysed and RNA extracted. Relative viral RNA amounts were determined by qRT-PCR with primers for the NP (segment 5) RNA and the host 18S rRNA. The means of two technical replicates are displayed as points, with the mean and standard deviation of three biological replicates indicated with whiskers. (**b**) esiRNA treated cells were transfected with VPOL, firefly luciferase minigenome, and *Renilla* luciferase expression vectors. Relative viral RNA replication was assessed by normalizing firefly luciferase activity to *Renilla* luciferase activity. Biological replicates are displayed as points, with the mean and standard deviation of six biological replicates indicated with whiskers. (**c** and **d**) At 12 h.p.i. cells were lysed and proteins extracted. Proteins were subjected to western blotting using antisera to (**c**) viral NP protein, or (**d**) viral M1 protein. Relative viral proteins in knockdown immunoblots were quantified by densitometry. After transfer, gels were stained with Coomassie Brilliant Blue; a section is shown as loading control

To understand the necessity for copper transport in earlier stages of infection, we analyzed viral RNA and protein syntheses in cells targeted by knockdown of CTR1 or ATP7A copper transporters (Fig. 3). CTR1 knockdown significantly reduced viral RNA synthesis (4 h.p.i, *p* = 0.006), as did ATP7A at this timepoint (*p* = 0.007). Viral RNA synthesis significantly lagged in CTR1 knockdown (12 h.p.i, *p* = 0.06; 24 h.p.i, *p* = 0.01), while knockdown of ATP7A did not produce a significant effect after 4 h.p.i. (Fig. 3a). These data suggested that copper distribution in the cell is necessary for viral RNA synthesis. To study whether copper transporters affect activity of the influenza A viral RNA-dependent RNA polymerase complex, we assayed viral polymerase activity in the absence of other viral processes, using a minigenome reporter assay (Fig. 3b). In alignment with reduced RNA synthesis during intact viral infection (Fig. 3a), viral polymerase activity was drastically reduced (*p* < 10^−7^) by knockdown of either CTR1 or ATP7A (Fig. 3b). In parallel to decreased viral RNA synthesis, in infected cells, synthesis of viral nucleoprotein (NP) (Fig. 3c) and matrix protein (M1) (Fig. 3d) were both markedly reduced by knockdown of either CTR1 or ATP7A. Interestingly, ATP7A knockdown caused more noticeable depression of viral RNA and protein synthesis than overall infectious particle production, suggesting that viral RNA and proteins are produced in excess in A/WSN/33 (H1N1) infection of A549 cells while copper transport is necessary for efficient virion production.

**Fig. 4:**
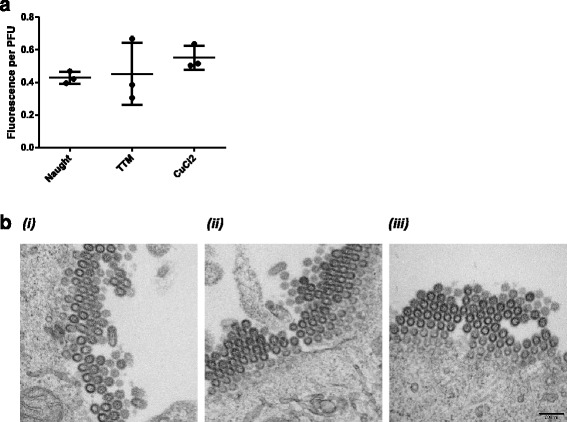
Alteration of copper does not disrupt virion neuraminidase activity or particle morphology. A549 cells were untreated (Naught) or treated 24 hours with 50 μM CuCl_2_ or 10 μM TTM before infection with influenza A/WSN/33 (H1N1) virus (MOI = 1). (**a**) Extracellular virus was harvested from cell supernatants 48 h.p.i. and analyzed by fluorescent substrate-based neuraminidase assay. Relative fluorescent units were normalized to the number of plaque forming units (PFU) in each sample. The means of 6 technical replicates are displayed as points, with the mean and standard deviation of three biological replicates indicated with whiskers. (**b**) Cells were fixed 12 h.p.i. for transmission electron microscopy (TEM) analysis of viral particles budding from plasma membrane; *(i)* no treatment (Naught), *(ii)* 10 μM TTM, *(iii)* 50 μM CuCl_2_. Scale bar, 200 nm

We found these results intriguing. In experiments where copper concentration was altered by copper chelator TTM , or adding exogenous CuCl_2_ a larger effect was observed on infectious particles released than on RNA and protein levels (Fig. 1). RNA and protein levels were however affected by knockdown of copper transporters, with concomitant reduced RNA (Fig. 3a) and protein (Fig. 3c, d) syntheses, upstream of an observed decrease in titer (Fig. 2a). Thus, we sought to further understand how changing total copper concentration might affect late steps in the viral lifecycle, *i.e.* virion assembly, maturation, and release. Neuraminidase (NA), the viral glycoprotein that facilitates release from the mother cell, undergoes maturation and export through the Golgi network. CuCl_2_ and TTM treatments likely affect copper concentrations within the Golgi, possibly affecting glycoprotein maturation and function such as the disulfide bonding required for NA function [37]. The amount of NA activity was assayed in particles released from cells treated with CuCl_2_ and TTM (Fig. 4a). A small increase in NA activity per infectious unit of virus was observed in particles produced by cells treated with exogenous CuCl_2_ (*p* = 0.03), but not with TTM copper chelator. This suggests that virion-associated neuraminidase enzyme activity is in part dependent on copper in the host cells. We have not ruled out a redox-related mechanism whereby exogenous CuCl_2_ treatment leading to excess Cu (I) in the cell (Table 1) could affect redox potential, and thus glycoprotein processing, in the secretory pathway. Future work will evaluate copper’s effect on the oligomerization/disulfide bond formation [38], protease cleavage [39], glycosylation [40], and trafficking of virion glycoproteins to the apical cell surface [41].

Also having observed reduced virus titers when copper concentrations were altered (Fig. 1), we hypothesized that virion assembly or morphology could be copper-dependent. To further analyze effects on assembly, the budding of virions from the plasma membrane was visualized. Cells were again treated with CuCl_2_ or TTM and infected with influenza A/WSN/33 (H1N1), then fixed and imaged by transmission electron microscopy (TEM). Although we had observed a quantifiable difference in infectious particle production under CuCl_2_ or TTM treatment (Fig. 1a), no significant differences in virion particle morphology and budding were noted (Fig. 4b). Spherical (100 nm diameter) and oblong (100-200 nm longitudinal axis) virus particles containing viral ribonucleoptoein (vRNP) segments were predominantly observed budding from untreated cells infected with A/WSN/33 (H1N1) virus (Fig. 4b), as well as a minority of filamentous particles (>200 nm longitudinal axis, <1%). In cells treated with CuCl_2_ or TTM prior to WSN infection, no observable differences were apparent in gross virion particle morphology. Thus, altering intracellular copper concentration apparently leads to less efficient virion formation (*i.e.,* lower titer), rather than a defect in virion morphology. NA activity is known to be a determinant of particle morphology, with increased neuraminidase activity observed on filamentous virions [42]. As virion NA activity was increased in exogenous copper treatment (Fig. 4a), we might also have expected a shift in the observed sphere-to-filament ratio. However, fewer distinct filamentous particles were visible budded from cells treated with CuCl_2_, although the difference in frequency was not significant. As influenza A/WSN/33 (H1N1) virus infection in cultured cells does not produce significant numbers of filamentous particles, further investigation of the possible role of copper in maturation of filamentous influenza A virions will require analyses of filamentous-producing virus, such as those harboring mutations in matrix proteins M1 [30, 42] or M2 [43].

**Fig. 5:**
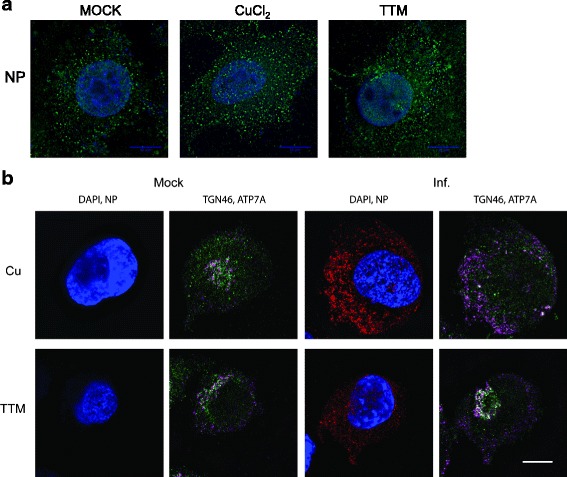
Localization of influenza nucleoprotein and copper transporter ATP7A in infected cells treated with exogenous copper of chelator. (**a**) A549 cells were untreated (Mock) or treated 24 hours with 50 μM CuCl_2_ or 10 μM TTM before infection with influenza A/WSN/33 (H1N1) virus (MOI = 1). Cells were fixed 12 h.p.i. for immunofluorescence microscopy to analyzing subcellular localization of NP. DAPI, blue; NP, green. Scale bar, 10 μm. (**b**) Immunofluorescence microscopy analyzing subcellular localization of ATP7A after CuCl_2_ or TTM at 12 h.p.i. DAPI, blue; TGN46, magenta; NP, red; ATP7A, green. Scale bar, 10 μm

To study how intracellular copper transport might regulate influenza virion proteins, viral nucleoprotein (NP) and copper transporter ATP7A were analyzed by immunofluorescence. Early in infection, NP can be found in the cell nucleus, later translocating to the cytoplasm with vRNP during virion assembly stages [7]. In concordance with intact virion assembly (Fig. 4b), infected cells showed little difference in punctate NP distribution in the cytoplasm at 12 h.p.i. under no treatment, exogenous CuCl_2_ or TTM treatments (Fig. 5a). As previously observed in other cell types [36], ATP7A is a membrane-associated transporter protein that localized to a vesicular cytoplasmic compartment that partly overlapped with *trans-*Golgi protein TGN46 (Fig. 2c), a pattern maintained after TTM treatment or exogenous CuCl_2_ treatment (Fig. 5b). While ATP7A did not fully co-localize with *trans*-Golgi marker TGN46, influenza A virus infection altered localization of ATP7A. ATP7A appeared in a dispersed vesicular pattern in the cytoplasm in infected cells treated with exogenous copper, but in a more concentrated pattern reminiscent of nuclear-proximal Golgi under TTM treatment (Fig. 5b). These results suggest that influenza A virus may disrupt copper-responsive trafficking of ATP7A in the cell secretory pathway. It is important to note that other viral proteins traffic through the cytoplasm during virion assembly. M2 functions to prevent acidification of Golgi lumen, which could prematurely trigger hemagglutinin (HA) conformational rearrangement [44, 45]. Copper is trafficked through the Golgi in a number of conditions, and HA glycoprotein processing or NA maturation could be affected by altered copper related conditions, in addition to potential effects on M2 as noted previously [27]. How intracellular copper distribution, and localization of copper transporter proteins including ATP7A might affect trafficking and maturation of viral proteins, and ultimately virus replication, is under further investigation.

**Fig. 6:**
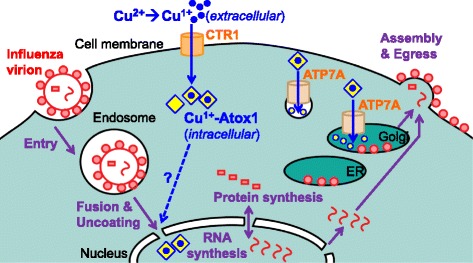
Model of copper-mediated regulation of the influenza virus life cycle. Extracellular copper [Cu^2+^] shares topological space with virion binding to host cell, and viral entry steps within the endosome. CTR1 imports extracellular copper to the cytoplasm. Intracellular copper [Cu^1+^] is associated with the ATOX1 chaperone and other metalloproteins. From there, copper is actively transported into the secretory pathway by ATP7A. ATP7A plays a role determining copper concentration in the cytosol and in ER, Golgi, and other membrane bound compartments, where the viral glycoproteins HA and NA (o) are synthesized and mature. New viral RNA are synthesized in the nucleus, where ATOX1 may transport intracellular [Cu^1+^]. In complex with matrix proteins (M1 and M2, ☐), genomic viral RNA progeny are exported from the nucleus to associate with M1, M2, HA, NA and other proteins to assemble budding virions at the plasma membrane, a site that is topologically in the cytosol

Cellular compartments where viral life cycle and copper transport overlap are summarized in Fig. 6. Entry of influenza particles into the cell, mediated by the HA, NA, and M2 proteins, involves interactions at the cell surface and fusion and uncoating from the endosome. These processes have the potential be affected as endocytosis can be affected by copper concentration [46], and ion concentrations are important for influenza’s entry steps [47]. Compartmentalized copper has been found to inhibit ion transport activity of viral M2 protein [27]; thus, intracellular copper distribution could also affect uncoating of entering virions. Viral RNA is synthesized in the cell nucleus, and viral proteins are synthesized in the cytosol. We found that the restriction of copper ions by knockdown of either CTR1 or ATP7A grossly affects viral RNA and protein syntheses in the nucleus and cytoplasm, respectively (Fig. 3). Intracellular copper distribution is tightly regulated by CTR1, importing copper into the cell, affecting the biological activity of chaperones such as ATOX1 that in turn regulate functions of cytoplasmic and nuclear host proteins, as well as ATP7A (Fig. 6). Disruption of the *trans*-Golgi copper importer ATP7A that regulates compartmentalized copper distribution may lead to accumulation of copper in the cytoplasm or disruption in other compartments (Fig. 6). However, increased expression of other copper transporters, for example ATP7B, may be induced when copper homeostasis is disrupted [48, 49], resulting in compensatory redistribution of intracellular copper. Thus, disruption of copper transport in infected cells may influence copper homeostasis, and the virus life cycle in a compartment-specific manner,

We expected the down-regulation of CTR1 to result in an intracellular environment similar to chelator (TTM) treatment, *i.e.* lower available copper. However, knockdown of CTR1 or ATP7A (Fig. 2 and Fig. 3) had a more dramatic effect on viral replication than the chelator (Fig. 1). CTR1 inhibition results in a mild reduction in available copper, and ATP7A knockdown results in copper accumulation in the cells (Table 1). Disruption of these proteins likely changes in copper availability and possibly redox potential in subcellular compartments. ATP7A transports copper ions from the cytosol to compartments including the Golgi network and vesicles, serving a biosynthetic role for the secretory pathway and copper export role to maintain cellular copper balance [48, 49]. It can be reasoned that in infected cells, ATP7A-mediated transport of Cu (I) is required for efficient viral RNA and viral protein syntheses (Fig. 3). We expected the effect of ATP7A knockdown would resemble that of increased exogenous CuCl_2_, leading to increased cytoplasmic copper but disrupted copper homeostasis in other cellular compartments that depend on ATP7A for copper import (Fig. 6). In parallel, maturation of viral glycoproteins and disulfide shuffling [50] occurs during through the Golgi network, a compartment dependent on ATP7A import of copper. Thus, initially we had expected an ATP7A-dependent viral replication defect might be at an entry or exit step. However, the results suggest ATP7A regulates synthesis of viral RNA and protein macromolecules, nuclear and cytosolic steps in the virus life cycle that are highly dependent on host factors [7].

## Conclusions

Viral processes are intimately entwined with host functions. Copper metabolism is important in a number of host functions, with diet and therapeutics under investigation for several genetic and developmental defects in rodent models [14, 51]. Here we show that cellular copper homeostasis is linked to the influenza A virus life cycle. This study showed that host proteins that transport copper into the cell (CTR1) and into the secretory pathway (ATP7A) are required for influenza A/WSN/33 (H1N1) RNA and protein syntheses. These data suggest that copper has specific roles in viral replication in the cytoplasm as well as in viral protein interactions with the secretory pathway. Further research into this relationship will help to define additional aspects of influenza processes, and may offer additional avenues for anti-influenza therapy development.

## Abbreviations

esiRNA: Endoribonuclease-prepared siRNA
h.p.i.: hours post infection
HA: Hemagglutinin
MOI: Multiplicity of infection
NA: Neuraminidase
NP: Nucleoprotein
ORF: Open reading frame
PBS: Phosphate buffered saline
qRT-PCR: quantitative real-time polymerase chain reaction
RNAi: RNA interference
siRNA: small interfering RNA
TEM: Transmission electron microscopy
TTM: tetrathiomolybdate
VPOL: Viral polymerase
vRNA: (influenza genomic) viral RNA
vRNP: viral ribonucleoprotein
